# Biological deep learning for simulating the role of SST Interneurons in cognitive resilience

**DOI:** 10.1002/alz70861_108740

**Published:** 2025-12-23

**Authors:** Benjamin Federico Elias Duisberg, Iris Marmouset‐De La Taille, Will Greedy, Becky C. Carlyle, Rui Ponte Costa

**Affiliations:** ^1^ University of Oxford, Oxford, Oxfordshire UK; ^2^ University of Bristol, Bristol, Bristol County UK

## Abstract

**Background:**

Two major snRNA‐seq studies have shown that SST interneurons specifically are lost along the AD disease trajectory (Gabitto et al., 2024, Mathys et al., 2023). We have previously shown that levels of SST‐14 peptide in the angular gyrus correlate with cognitive performance independent of AD pathology (Morgan & Carlyle, 2024). To explore why SST is a key predictor of cognitive decline, we used a state‐of‐the‐art cortical network model that approximates both deep learning and a large number of neuroscience studies (BurstCCN, Greedy et al. 2022).

**Methods:**

We simulated progressive SST‐cell degeneration by permanently removing SST‐like inhibitory connections in BurstCCN, and measured "cognitive performance" as the network’s ability to learn and generalize a digit classification task (MNIST). For comparison, we also assessed the impact of removing pyramidal‐like excitatory connections.

**Results:**

The loss of SST‐like connections led to a substantially greater reduction in test accuracy than the loss of pyramidal‐like connections, highlighting the importance of SST‐mediated inhibition in network function. This decline was primarily due to disrupted feedback input to the apical dendrites of pyramidal neurons, impairing hierarchical processing. Notably, our simulation results aligned with single‐nucleus RNA sequencing data from post‐mortem brains of AD patients, showing a correlation between SST cell abundance and cognitive resilience across individuals with varying levels of neuropathology.

**Conclusion:**

Our findings suggest that SST interneuron loss has a disproportionately large impact on cognitive function, both in artificial and biological systems. The model predicts that this is due to the key role that SST interneurons play in propagating task‐error signals throughout the cortex. These results not only support the hypothesis that SST interneurons play a critical role in cognitive resilience, but also provide deeper insight into Alzheimer's disease by linking cortical circuit physiology to cognitive dysfunction.

**References**

Gabitto, M. I. et al. Nature Neuroscience (2024).

Mathys, H. et al. Cell (2023).

Morgan, G. R. & Carlyle, B. C. Scientific Reports (2024).

Greedy, W. et al. Advances in neural information processing systems (2022).

